# Supplementary material for: Beyond aging, sex, and insomnia disorder shape NREM brain oscillations
Source: Sleep. 2026 Jul 15;49(10):zsag192. doi: 10.1093/sleep/zsag192 (PMC13658286; doi:10.1093/sleep/zsag192)
Supplement: Supplementary file 1 [file supplemental_material_2026_07_13_zsag192.docx]

**Beyond Aging, Sex and Insomnia Disorder Shape NREM Brain Oscillations**

Nyissa A. Walsh^1,2,3,4,5^ Aurore A. Perrault^1,4,6,^ Nathan E. Cross^1,4,7,^ Antonia Maltezos^1,3,4,5^ Emma-Maria Phillips^1,4,5,8^, Loïc Barbaux^1,3,4,5^, Oren Weiner^1,2,3,4,5^ Claire Dyment^1,2,4,5,9^ Florence Borgetto^1,3,4,5^ Jean-Phillipe Gouin^2,4,5,10^ & Thien Thanh Dang-Vu^1,3,4,5^

1. Sleep, Cognition, & Neuroimaging Lab, Department of Health, Kinesiology, & Applied Physiology, Concordia University, Montreal, Canada

2. Department of Psychology, Concordia University, Montreal, Canada

3. Centre for Studies in Behavioral Neurobiology, Concordia University, Montreal, Canada

4. Centre de Recherche de l’Institut Universitaire de Gériatrie de Montreal, Canada

5. School of Health, Concordia University, Montreal, Canada

6. Woolcock Institute of Medical Research, Macquarie University, Sydney, Australia

7. School of Psychology, Faculty of Science, University of Sydney, Australia

8. Department of Neurosciences, Faculty of Medicine, Université de Montréal, Montréal, QC, Canada

9. Institute of Psychiatry, Psychology and Neuroscience, King’s College London, United Kingdom

10. Stress, Interpersonal Relationships and Health Laboratory, Department of Psychology, Concordia University, Montreal, Canada

*Corresponding authors: [nyissa.walsh@mail.concordia.ca](mailto:nyissa.walsh@mail.concordia.ca), tt.dangvu@concordia.ca

Concordia University, 7141 Rue Sherbrooke O, H4B 1R6, Montreal, QC, Canada

Telephone: +1 (514) 848-2424, x4037

Fax: N/A

**Supplemental Material**

Supplemental Table 1 – *Age Differences in Sleep Macroarchitecture Variables*

|  | **Wald χ² (df= 1)** | **p** | **B** | **β** | **SE** | ***p*** | **95% Lower** | **Upper** | **Difference** |
| --- | --- | --- | --- | --- | --- | --- | --- | --- | --- |
| **TIB (min)** | 0.56 | .445 | 0.05 | 0.06 | 0.23 | .807 | -.410 | .486 | - |
| **TST (min)** | 16.06 | < .001 | **-0.87** | **-0.24** | **0.23** | **<.001** | **-1.34** | **-0.46** | **↓** |
| **SOL (min)** | 0.12 | .730 | 0.03 | 0.03 | 0.08 | .757 | -0.11 | 0.18 | - |
| **WASO (min)** | 33.89 | < .001 | **0.77** | **0.37** | **0.13** | **<.001** | **0.51** | **1.03** | **↑** |
| **SE (%)** | 41.53 | < .001 | **-0.22** | **-0.40** | **0.04** | **<.001** | **-0.29** | **-0.16** | **↓** |
| **Wake (%)** | 37.71 | < .001 | **0.19** | **0.38** | **0.03** | **<.001** | **0.13** | **0.25** | **↑** |
| **NREM1 (%)** | 0.17 | .678 | 0.01 | 0.07 | 0.02 | .696 | -0.02 | 0.04 | - |
| **NREM2 (%)** | 11.78 | < .001 | **-0.39** | **-0.16** | **0.11** | **.005** | **-0.62** | **-0.17** | **↓** |
| **NREM3 (%)** | 34.27 | < .001 | **-0.42** | **-0.31** | **0.07** | **<.001** | **-0.57** | **-0.29** | **↓** |
| **REM (%)** | 16.99 | < .001 | **-0.27** | **-0.21** | **0.07** | **.002** | **-0.40** | **-0.14** | **↓** |
| **SFI** | 42.24 | < .001 | **0.09** | **0.56** | **0.01** | **<.001** | **0.06** | **0.12** | **↑** |

Note. Bias-corrected confidence intervals are presented. B= unstandardized coefficient, β = standardized coefficient, *p* = adjusted p. Significant results are bolded. TIB = time in bed; TST = total sleep time; SOL = sleep onset latency; WASO = wake after sleep onset; SE= Sleep efficiency; NREM = non-rapid eye movement sleep; REM = rapid eye movement sleep; SFI = sleep fragmentation index;

Supplemental Table 2 – *Age Controlled Group Differences in Sleep Macroarchitecture Variables*

|  | **INS (n= 119)** | **HS (n= 103)** | **Wald χ² (df= 1)** | **p** | **B** | **β** | **SE** | ***p*** | **95% Lower** | **Upper** | **Difference** |
| --- | --- | --- | --- | --- | --- | --- | --- | --- | --- | --- | --- |
| **TIB (min)** | 445.67 ± 5.31 | 453.51 ± 5.27 | 1.11 | .292 | -7.84 | 0.13 | 7.52 | .308 | -22.71 | 6.35 | **-** |
| **TST (min)** | 364.51 ± 5.71 | 398.81 ± 4.84 | 20.96 | < .001 | **-34.30** | **0.55** | **7.53** | **<.001** | **-48.15** | **-18.97** | **INS < HS** |
| **SOL (min)** | 14.89 ± 1.31 | 13.93 ± 1.54 | 0.21 | .645 | 0.96 | 0.06 | 2.14 | .650 | -3.78 | 4.92 | - |
| **WASO (min)** | 58.80 ± 3.85 | 34.67 ± 2.15 | 27.80 | < .001 | **24.14** | **0.63** | **4.60** | **<.001** | **15.23** | **32.94** | **INS > HS** |
| **SE (%)** | 81.85 ± 0.93 | 88.16 ± 0.61 | 30.21 | < .001 | **-6.30** | **0.63** | **1.14** | **<.001** | **-8.64** | **-4.09** | **INS < HS** |
| **Wake (%)** | 13.83 ± 0.90 | 7.95 ± 0.47 | 31.39 | < .001 | **5.88** | **0.65** | **1.05** | **<.001** | **3.92** | **8.00** | **INS > HS** |
| **NREM1 (%)** | 3.23 ± 0.21 | 5.03 ± 0.43 | 12.30 | <.001 | **-1.80** | **-0.53** | **0.52** | **.007** | **-2.93** | **-0.82** | **INS <HS** |
| **NREM2 (%)** | 44.41 ± 1.15 | 64.05 ± 4.95 | 18.72 | < .001 | **-19.65** | **-0.47** | **4.61** | **.005** | **-29.11** | **-11.13** | **INS < HS** |
| **NREM3 (%)** | 20.67 ± 0.87 | 31.93 ± 2.58 | 21.24 | < .001 | **-11.26** | **-0.47** | **2.35** | **<.001** | **-16.36** | **-6.78** | **INS < HS** |
| **REM (%)** | 22.90 ± 0.70 | 31.06 ± 2.58 | 11.57 | < .001 | **-8.16** | **-0.37** | **2.42** | **.007** | **-13.07** | **-3.45** | **INS < HS** |
| **SFI** | 7.66 ± 0.23 | 7.51 ± 0.27 | 0.16 | .686 | 0.15 | 0.08 | 0.37 | .676 | -0.59 | 0.85 | - |

Note. Values are reported as estimated marginals means (age controlled) ± standard error with bias-corrected confidence intervals. B= unstandardized coefficient, β = standardized coefficient, *p* = adjusted p. Significant results are bolded. TIB = time in bed; TST = total sleep time; SOL = sleep onset latency; WASO = wake after sleep onset; SE= Sleep efficiency; NREM = non-rapid eye movement sleep; REM = rapid eye movement sleep; SFI = sleep fragmentation index; INS = insomnia group; HS = healthy sleeper group.

Supplemental Table 3 – *Age Controlled Sex Differences in Sleep Macroarchitecture Variables*

|  | **Male (n= 74)** | **Female (n= 148)** | **Wald χ² (df= 1)** | **p** | **B** | **β** | **SE** | ***p*** | **95% Lower** | **Upper** | **Difference** |
| --- | --- | --- | --- | --- | --- | --- | --- | --- | --- | --- | --- |
| **TIB (min)** | 458.03 ± 6.30 | 444.95 ± 4.79 | 2.64 | .104 | 13.08 | 0.23 | 8.11 | .106 | -2.45 | 29.22 | **-** |
| **TST (min)** | 383.28 ± 6.35 | 379.00 ± 5.09 | 0.27 | .604 | 4.28 | 0.04 | 8.14 | .601 | 11.66 | 20.05 | **-** |
| **SOL (min)** | 13.31 ± 1.36 | 15.02 ± 1.24 | 0.90 | .343 | -1.70 | 0.12 | 1.80 | .348 | -5.40 | 1.92 | **-** |
| **WASO (min)** | 53.99 ± 3.57 | 44.41 ± 2.91 | 4.49 | .034 | **9.58** | **0.28** | **4.65** | **.041** | **0.31** | **18.57** | **M > F** |
| **SE (%)** | 83.73 ± 0.93 | 85.30 ± 0.74 | 1.78 | .182 | -1.57 | 0.19 | 1.15 | .173 | -3.82 | 0.77 | **-** |
| **Wake (%)** | 12.39 ± 0.79 | 10.46 ± 0.69 | 3.51 | .061 | 1.93 | 0.24 | 1.05 | .059 | -0.10 | 4.01 | **-** |
| **NREM1 (%)** | 5.15 ± 0.47 | 3.53 ± 0.21 | 9.94 | .002 | **1.62** | **0.48** | **0.49** | **.004** | **0.70** | **2.59** | **M > F** |
| **NREM2 (%)** | 56.68 ± 5.35 | 51.94 ± 3.16 | 0.56 | .454 | 4.75 | 0.09 | 6.28 | .452 | -7.39 | 17.93 | - |
| **NREM3 (%)** | 26.25 ± 3.14 | 25.72 ± 1.55 | 0.23 | .880 | 0.53 | 0.003 | 3.42 | .873 | -5.83 | 7.70 | - |
| **REM (%)** | 27.65 ± 2.79 | 26.20 ± 1.65 | 0.19 | .661 | 1.45 | 0.05 | 3.28 | .659 | -4.64 | 8.01 | - |
| **SFI** | 8.60 ± 0.24 | 7.09 ± 0.21 | 24.43 | < .001 | **1.52** | **0.51** | **0.31** | **< .001** | **0.93** | **2.13** | **M > F** |

Note. Values are reported as estimated marginals means (age controlled) ± standard error with bias-corrected confidence intervals. B= unstandardized coefficient, β = standardized coefficient*, p* = adjusted p. Significant results are bolded. Sigma power (11.25-16Hz), SWA (0.25-4Hz), SO power (0.25-1.25 Hz), NREM2 + NREM3, M = male; F = female.

Supplemental Table 4- *Age Controlled* *GroupxSex Differences in Sleep Macroarchitecture Variables*

| **Variable** | **Male INS**  **(n= 34)** | **Female INS (n= 85)** | **Male HS**  **(n= 40)** | **Female HS**  **(n= 63)** | **Wald χ² (df)** | **p** | **B** | **β** | **SE** | ***p*** | **95% Lower** | **Upper** | **Difference** |
| --- | --- | --- | --- | --- | --- | --- | --- | --- | --- | --- | --- | --- | --- |
| **Age (yrs)** | 41.29 ± 2.34 | 49.76 ± 1.58 | 34.00 ± 2.83 | 35.78 ± 2.32 | 38.79 (3) | <.001 | 8.47 | 0.48 | 2.82 | .005 | 2.33 | 14.25 | FINS > MINS |
|  |  |  |  |  |  |  | 13.99 | 0.80 | 2.72 | <.001 | 8.60 | 19.48 | FINS > FHS |
|  |  |  |  |  |  |  | -15.77 | -0.90 | 3.18 | <.001 | -22.05 | -9.23 | FINS > MHS |
|  |  |  |  |  |  |  | -7.29 | -0.41 | 3.67 | .039 | -14.30 | -0.06 | MINS > MHS |
| **TIB (min)** | 450.07 ± 9.95 | 443.63 ± 6.26 | 465.21 ± 7.70 | 446.47 ± 7.10 | 4.96 (3) | .175 | 21.58 | 0.34 | 10.32 | .028 | 3.19 | 40.49 | FINS < MHS |
| **TST (min)** | 360.71 ± 9.86 | 366.12 ± 7.07 | 404.92 ± 6.31 | 394.82 ± 6.81 | 25.10 (3) | <.001 | -44.21 | -0.81 | 11.68 | <.001 | -66.51 | -22.17 | MINS < MHS |
|  |  |  |  |  |  |  | -34.11 | -0.62 | 11.99 | .010 | -58.82 | -9.77 | MINS < FHS |
|  |  |  |  |  |  |  | 28.70 | 0.66 | 9.84 | .004 | 8.46 | 48.87 | FINS < FHS |
|  |  |  |  |  |  |  | -38.80 | -0.84 | 9.72 | <.001 | -57.03 | -20.34 | FINS < MHS |
| **SOL (min)** | 14.46 ± 1.96 | 15.10 ± 1.63 | 12.29 ± 1.94 | 14.93 ± 2.03 | 1.50 (3) | .682 | - | - | - | - | - | - | - |
| **WASO (min)** | 64.27 ± 6.49 | 56.32 ± 4.67 | 43.30 ± 2.69 | 29.56 ± 2.56 | 43.48 (3) | <.001 | -26.74 | -0.97 | 5.73 | <.001 | -38.99 | -15.57 | FINS > FHS |
|  |  |  |  |  |  |  | 13.03 | 0.66 | 5.78 | .032 | 2.49 | 24.25 | FINS > MHS |
|  |  |  |  |  |  |  | 20.97 | 0.69 | 7.30 | .008 | 6.93 | 36.98 | MINS > MHS |
|  |  |  |  |  |  |  | 34.68 | 1.00 | 6.91 | <.001 | 21.78 | 48.86 | MINS > FHS |
|  |  |  |  |  |  |  | -13.71 | -0.31 | 3.59 | <.001 | -20.30 | -7.14 | MHS > FHS |
| **SE (%)** | 80.18 ± 1.55 | 82.59 ± 1.15 | 87.23 ± .74 | 88.65 ± .81 | 33.83 (3) | <.001 | 7.05 | 0.86 | 1.73 | <.001 | 5.15 | 12.36 | MINS < MHS |
|  |  |  |  |  |  |  | 8.46 | 0.96 | 1.76 | <.001 | 5.15 | 12.36 | MINS < FHS |
|  |  |  |  |  |  |  | -6.05 | -0.92 | 1.40 | <.001 | -8.80 | -3.28 | FINS < FHS |
|  |  |  |  |  |  |  | -4.63 | -0.82 | 1.44 | .004 | -7.45 | -2.25 | FINS < MHS |
| **Wake (%)** | 15.07 ± 1.41 | 13.26 ± 1.12 | 9.63 ± .56 | 6.97 ± .59 | 44.30 (3) | <.001 | -6.29 | -0.98 | 1.34 | <.001 | -8.89 | -3.90 | FINS > FHS |
|  |  |  |  |  |  |  | -3.63 | -0.73 | 1.33 | .014 | -6.39 | -1.29 | FINS > MHS |
|  |  |  |  |  |  |  | 5.44 | 0.75 | 1.55 | .002 | 2.50 | 8.29 | MINS > MHS |
|  |  |  |  |  |  |  | 8.10 | 1.00 | 1.51 | <.001 | 4.82 | 11.25 | MINS > FHS |
|  |  |  |  |  |  |  | 2.66 | 0.25 | 0.75 | <.001 | 1.16 | 4.15 | MHS > FHS |
| **NREM1** | 3.84 ± .41 | 2.95 ± .24 | 6.37 ± .75 | 4.22 ± .44 | 19.59 (3) | <.001 | -3.42 | 1.01 | 0.88 | <.001 | -5.19 | -1.88 | FINS < MHS |
| **(%)** |  |  |  |  |  |  | -1.27 | 0.35 | 0.53 | .049 | -2.45 | -0.19 | FINS < FHS |
|  |  |  |  |  |  |  | 2.15 | 0.66 | 0.80 | .013 | 0.58 | 3.79 | FHS < MHS |
|  |  |  |  |  |  |  | 2.54 | 0.76 | 0.82 | .009 | 0.99 | 4.02 | MINS < MHS |
| **NREM2** | 43.38 ± 1.70 | 44.80 ± 1.48 | 69.38 ± 9.55 | 60.69 ± 6.05 | 19.47 (3) | <.001 | 15.89 | 0.51 | 5.68 | .022 | 5.97 | 27.83 | FINS < FHS |
| **(%)** |  |  |  |  |  |  | 24.57 | 0.74 | 9.41 | .025 | 8.80 | 43.16 | FINS < MHS |
|  |  |  |  |  |  |  | -26.00 | -0.69 | 10.12 | .037 | -47.06 | -7.38 | MINS < MHS |
|  |  |  |  |  |  |  | -15.89 | -0.47 | 5.67 | .020 | -27.82 | -5.17 | MINS < FHS |
| **NREM3 (%)** | 15.96 ± 1.18 | 22.70 ± 1.10 | 35.71 ± 5.52 | 29.33 ± 2.80 | 33.84 (3) | <.001 | 6.74 | 0.13 | 1.63 | <.001 | 3.58 | 9.92 | MINS < FINS |
|  |  |  |  |  |  |  | 19.75 | 0.97 | 5.89 | .009 | 9.24 | 31.83 | MINS < MHS |
|  |  |  |  |  |  |  | -13.37 | -0.67 | 3.11 | <.001 | -20.35 | -7.74 | MINS < FHS |
|  |  |  |  |  |  |  | -6.64 | -0.54 | 2.77 | .024 | -12.62 | -1.47 | FINS < FHS |
|  |  |  |  |  |  |  | -13.01 | -0.84 | 5.45 | .035 | -25.29 | -2.92 | FINS < MHS |
| **REM (%)** | 21.42 ± 1.29 | 23.52 ± .84 | 33.50 ± 4.96 | 29.46 ± 3.17 | 11.80 (3) | .008 | -8.04 | -0.44 | 3.38 | .042 | -14.94 | -1.67 | MINS < FHS |
|  |  |  |  |  |  |  | 12.07 | 0.64 | 5.20 | .035 | 3.05 | 22.89 | MINS < MHS |
|  |  |  |  |  |  |  | **-5.95** | **-0.45** | **2.94** | **.057** | **-12.31** | **-0.32** | **FINS < FHS** |
|  |  |  |  |  |  |  | -9.98 | -0.65 | 5.01 | .07 | -21.55 | -1.21 | FINS < MHS |
| **SFI** | 8.77 ± .29 | 7.17 ± .30 | 8.45 ± .36 | 6.99 ± .32 | 27.13 (3) | <.001 | 1.78 | 0.77 | 0.45 | <.001 | 0.94 | 2.65 | MINS > FHS |
|  |  |  |  |  |  |  | -1.28 | -0.07 | 0.49 | .012 | -2.28 | -0.31 | FINS < MHS |
|  |  |  |  |  |  |  | 1.46 | 0.43 | 0.45 | <.001 | 0.61 | 2.32 | MHS > FHS |

Note. Values are reported as mean ± standard errors with bias-corrected confidence intervals. Only significant post-hoc analyses are presented. B= unstandardized coefficient, β = standardized coefficient*, p* = adjusted p; Results in bold are FDR-corrected comparisons p > .05 but became significant after bias-corrected confidence intervals. INS = insomnia group; HS = healthy sleeper group; TIB = time in bed; TST = total sleep time; SOL = sleep onset latency; WASO = wake after sleep onset; SE= Sleep efficiency; NREM = non-rapid eye movement sleep; REM = rapid eye movement sleep; SFI = sleep fragmentation index;

Supplemental Table 5- *Raw Means for Measures of Sleep Spindle and SWA (Fz)*

| **Variable (Fz)** | **INS**  **(n= 119)** | **HS**  **(n= 103)** | **Male**  **(n= 74)** | **Female**  **(n= 148)** | **Male INS**  **(n= 34)** | **Female INS**  **(n= 85)** | **Male HS**  **(n= 40)** | **Female HS**  **(n= 63)** |
| --- | --- | --- | --- | --- | --- | --- | --- | --- |
| **Event Detection** | | | | | | | | |
| Spindle Density | 2.07 ± .05 | 3.04 ± .084 | 2.54 ± .09 | 2.51 ± .71 | 2.16 ± .09 | 2.04 ± .05 | 2.87 ± .13 | 3.14 ± .11 |
| Spindle Amplitude | 104.50 ± 2.27 | 121.86 ± 4.34 | 106.46 ± 4.02 | 115.60 ± 3.00 | 99.04 ± 3.65 | 106.69 ± 2.80 | 112.78 ± 6.65 | 127.63 ± 5.62 |
| SO Density | 5.09 ± .09 | 7.92 ± .37 | 6.59 ± .38 | 6.31 ± .24 | 5.00 ± .19 | 5.13 ± .10 | 7.94 ± .62 | 7.90 ± .47 |
| SO Amplitude | 143.02 ± 3.86 | 182.24 ± 7.10 | 150.83 ± 6.65 | 166.41 ± 5.13 | 133.02 ± 5.92 | 147.01 ± 4.81 | 165.97 ± 10.73 | 192.57 ± 9.23 |
| **Power Spectral Analysis** | | | | | | | | |
| Absolute Sigma Power | 12.40 ± .64 | 13.71 ± .94 | 11.70 ± .80 | 13.62 ± .72 | 12.50 ± 1.25 | 12.36 ± .75 | 10.97 ± 1.02 | 15.40 ± 1.34 |
| Absolute SWA | 538.81 ± 30.32 | 817.43 ± 57.33 | 558.92 ± 43.30 | 715.40 ± 42.20 | 442.87 ± 36.13 | 577.18 ± 39.26 | 665.37 ± 72.36 | 911.21 ± 79.28 |
| Absolute SO Power | 436.71 ± 27.34 | 673.09 ± 51.37 | 481.04 ± 49.65 | 573.13 ± 34.88 | 356.30 ± 32.61 | 468.87 ± 35.50 | 595.67 ± 86.83 | 720.84 ± 63.26 |
| Relative Sigma Power | .020 ± .001 | .029 ± .001 | .027 ± .001 | .023 ± .001 | .024 ± .002 | .019 ± .001 | .031 ± .001 | .028 ± .001 |
| Relative SWA | .78 ± .01 | .80 ± .01 | .79 ± .01 | .80 ± .01 | .78 ± .01 | .79 ± .01 | .79 ± .01 | .81 ± .01 |
| Relative SO Power | .62 ± .01 | .64 ± .01 | .63 ± .01 | .63 ± .01 | .61 ± .02 | .62 ± .01 | .65 ± .02 | .63 ± .01 |

Note. Values are reported as raw mean ± standard error. INS = insomnia group; HS = healthy sleeper group. Sigma power (11.25-16Hz), SWA (0.25-4Hz), SO power (0.25-1.25 Hz). NREM2 + NREM3

Supplemental Table 6- *Raw Means for Measures of Sleep Spindle and SWA (Cz)*

| **Variable (Cz)** | **INS**  **(n= 119)** | **HS**  **(n= 103)** | **Male**  **(n= 74)** | **Female**  **(n= 148)** | **Male INS**  **(n= 34)** | **Female INS**  **(n= 85)** | **Male HS**  **(n= 40)** | **Female HS**  **(n= 63)** |
| --- | --- | --- | --- | --- | --- | --- | --- | --- |
| **Event Detection** | | | | | | | | |
| Spindle Density | 2.24 ± .06 | 3.27 ± .09 | 2.80 ± .10 | 2.68 ± .08 | 2.38 ± .12 | 2.19 ± .07 | 3.15 ± .14 | 3.34 ± .12 |
| Spindle Amplitude | 97.10 ± 2.27 | 107.95 ± 2.77 | 91.81 ± 2.31 | 107.30 ± 2.34 | 88.47 ± 2.79 | 100.56 ± 2.90 | 94.65 ± 3.52 | 116.39 ± 3.57 |
| SO Density | 5.00 ± .09 | 7.83 ± .38 | 6.48 ± .39 | 6.22 ± .24 | 4.95 ± .19 | 5.01 ± .11 | 7.78 ± .63 | 7.86 ± .49 |
| SO Amplitude | 129.90 ± 3.18 | 155.83 ± 4.67 | 129.05 ± 4.00 | 148.36 ± 3.74 | 119.14 ± 4.58 | 134.20 ± 3.97 | 137.48 ± 6.02 | 167.47 ± 6.21 |
| **Power Spectral Analysis** | | | | | | | | |
| Absolute Sigma Power | 15. 69 ± .78 | 17.05 ± 1.00 | 14.28 ± .95 | 17.29 ± .79 | 14.98 ± 1.41 | 15.98 ± .94 | 13.64 ± 1.28 | 19.16 ± 1.35 |
| Absolute SWA | 417.01 ± 22.43 | 647.44 ± 42.75 | 440.29 ± 35.44 | 559.76 ± 30.95 | 340.89 ± 25.77 | 447.46 ± 29.10 | 531.64 ± 60.36 | 718.86 ± 56.67 |
| Absolute SO Power | 324.76 ± 20.48 | 543.66 ± 48.39 | 390.25 ± 55.15 | 439.12 ± 26.83 | 261.29 ± 23.57 | 350.15 ± 26.66 | 508.76 ± 100.32 | 565.17 ± 48.50 |
| Relative Sigma Power | .031 ± .001 | .025 ± .001 | .029 ± .002 | .028 ± .001 | .034 ± .002 | .029 ± .001 | .025 ± .002 | .026 ± .002 |
| Relative SWA | .75 ± .01 | .77 ± .01 | .75 ± .01 | .76 ± .01 | .75 ± .01 | .75 ± .01 | .76 ± .013 | .78 ± .01 |
| Relative SO Power | .56 ± .01 | .60 ± .01 | .58 ± .01 | .58 ± .01 | .56 ± .02 | .56 ± .01 | .61 ± .02 | .59 ± .01 |

Note. Values are reported as raw mean ± standard error. INS = insomnia group; HS = healthy sleeper group. Sigma power (11.25-16Hz), SWA (0.25-4Hz), SO power (0.25-1.25 Hz). NREM2 + NREM3

Supplemental Table 7- *Raw Means for Measures of Sleep Spindle and SWA (Pz)*

| **Variable (Pz)** | **INS**  **(n= 119)** | **HS**  **(n= 103)** | **Male**  **(n= 74)** | **Female**  **(n= 148)** | **Male INS**  **(n= 34)** | **Female INS**  **(n= 85)** | **Male HS**  **(n= 40)** | **Female HS**  **(n= 63)** |
| --- | --- | --- | --- | --- | --- | --- | --- | --- |
| **Event Detection** | | | | | | | | |
| Spindle Density | 2.46 ± .62 | 3.58 ± .11 | 3.06 ± .12 | 2.93 ± .09 | 2.60 ± .11 | 2.40 ± .07 | 3.45 ± .17 | 3.66 ± .14 |
| Spindle Amplitude | 79.46 ± 1.75 | 96.32 ± 3.88 | 80.92 ± 4.13 | 90.46 ± 2.35 | 72.30 ± 2.30 | 82.33 ± 2.21 | 88.26 ± 7.23 | 101.44 ± 4.31 |
| SO Density | 4.71 ± .09 | 7.35 ± .36 | 6.06 ± .37 | 5.87 ± .23 | 4.59 ± .19 | 4.76 ± .10 | 7.31 ± .60 | 7.37 ± .46 |
| SO Amplitude | 110.51 ± 2.83 | 139.49 ± 5.63 | 112.65 ± 5.40 | 129.61± 3.84 | 100.57 ± 4.18 | 114.48 ± 3.52 | 122.91 ± 9.07 | 150.01 ± 6.92 |
| **Power Spectral Analysis** | | | | | | | | |
| Absolute Sigma Power | 13.98 ± .77 | 14.66 ± .87 | 12.41 ± .90 | 15.20 ± .72 | 12.71 ± 1.17 | 14.49 ± .97 | 12.14 ± 1.37 | 16.21 ± 1.09 |
| Absolute SWA | 318.11 ± 18.08 | 467.81 ± 33.90 | 309.71 ± 26.07 | 422.36 ± 24.47 | 247.00 ± 18.96 | 346.55 ± 23.51 | 367.34 ± 45.18 | 529.76 ± 45.66 |
| Absolute SO Power | 278.07 ± 18.91 | 431.12 ± 39.57 | 303.06 ± 43.43 | 368.22 ± 23.24 | 213.60 ± 20.52 | 303.86 ± 24.69 | 385.27 ± 79.32 | 459.39 ± 41.42 |
| Relative Sigma Power | .036 ± .001 | .038 ± .001 | .041 ± .002 | .035 ± .001 | .040 ± .003 | .034 ± .002 | .042 ± .002 | .036 ± .002 |
| Relative SWA | .72 ± .01 | .74 ± .01 | .72 ± .01 | .74 ± .01 | .72 ± .01 | .72 ± .01 | .73 ± .01 | .75 ± .01 |
| Relative SO Power | .60 ± .01 | .64 ± .01 | .62 ± .01 | .62 ± .01 | .59 ± .02 | .61 ± .01 | .65 ± .02 | .63 ± .01 |

Note. Values are reported as raw mean ± standard error. INS = insomnia group; HS = healthy sleeper group. Sigma power (11.25-16Hz), SWA (0.25-4Hz), SO power (0.25-1.25 Hz). NREM2 + NREM3

Supplemental Table 8- *Age Differences in Sigma and SWA Across All Participants*

| **Variable** | **Wald χ² (df= 1)** | **p** | **Coefficient** | **SE** | ***p*** | **95% Lower** | **Upper** | **Difference** |
| --- | --- | --- | --- | --- | --- | --- | --- | --- |
| **Fz** | | | | | | | | |
| **Event Detection** | | | | | | | | |
| Spindle Amplitude | 82.37 | <.001 | **-0.99** | **0.107** | **< .001** | **-1.20** | **-0.78** | **↓** |
| SO Amplitude | 122.93 | .000 | **-1.95** | **0.185** | **< .001** | **-2.35** | **-1.58** | **↓** |
| **Power Spectral Analysis** | | | | | | | | |
| Relative SO power | 27.26 | < .001 | **-0.002** | **0.001** | **<.001** | **-0.003** | **-0.001** | **↓** |
| Absolute Sigma power | 47.48 | < .001 | **-0.195** | **.029** | **<.001** | **-0.253** | **-0.138** | **↓** |
| Absolute SWA | 92.28 | < .001 | **-14.33** | **1.54** | **< .001** | **-17.57** | **-11.24** | **↓** |
| Absolute SO power | 70.89 | < .001 | **-12.00** | **1.43** | **< .001** | **-14.97** | **-9.05** | **↓** |
| **Cz** | | | | | | | | |
| **Event Detection** | | | | | | | | |
| Spindle Amplitude | 88.35 | .000 | **-0.76** | **0.08** | **< .001** | **-0.92** | **-0.62** | **↓** |
| SO Amplitude | 131.24 | .000 | **-1.44** | **0.13** | **< .001** | **-1.70** | **-1.19** | **↓** |
| **Power Spectral Analysis** | | | | | | | | |
| Relative SO power | 36.10 | < .001 | **-0.003** | **0.000** | **<.001** | **-0.004** | **-0.002** | **↓** |
| Absolute Sigma power | 42.18 | < .001 | **-0.206** | **.033** | **< .001** | **-0.270** | **-0.139** | **↓** |
| Absolute SWA | 98.75 | < .001 | **-10.81** | **1.09** | **< .001** | **-13.06** | **-8.60** | **↓** |
| Absolute SO power | 65.35 | < .001 | **-9.64** | **1.17** | **< .001** | **-11.82** | **-7.49** | **↓** |
| **Pz** | | | | | | | | |
| **Event Detection** | | | | | | | | |
| Spindle Density | 10.31 | < .001 | **-0.01** | **0.004** | **.003** | **-0.02** | **-0.004** | **↓** |
| Spindle Amplitude | 63.12 | <.001 | **-0.71** | **0.089** | **< .001** | **-0.91** | **-0.56** | **↓** |
| SO Density | 2.15 | .142 | -0.02 | 0.011 | .160 | -0.037 | 0.006 | - |
| SO Amplitude | 109.73 | .000 | **-1.40** | **0.136** | **< .001** | **-1.69** | **-1.15** | **↓** |
| **Power Spectral Analysis** | | | | | | | | |
| Relative Sigma Power | 2.38 | .123 | 8.65 × 10^-5^ | 5.71 × 10^-5^ | .126 | -2.29 × 10^-5^ | 0.000 | - |
| Relative SWA | 39.29 | < .001 | **-0.002** | **.000** | **< .001** | **-0.002** | **-0.001** | **↓** |
| Relative SO power | 30.35 | < .001 | **-0.002** | **.000** | **<.001** | **-0.003** | **-0.002** | **↓** |
| Absolute Sigma power | 23.39 | <.001 | **-0.141** | **.029** | **<.001** | **-0.203** | **-0.077** | **↓** |
| Absolute SWA | 69.69 | < .001 | **-7.49** | **.926** | **< .001** | **-9.22** | **-5.89** | **↓** |
| Absolute SO power | 46.71 | < .001 | **-7.01** | **1.05** | **< .001** | **-9.28** | **-4.91** | **↓** |

Note. Bias-corrected confidence intervals are presented. *p* = adjusted p; Significant results are bolded. Sigma power (11.25-16Hz), SWA (0.25-4Hz), SO power (0.25-1.25 Hz). NREM2 + NREM3

Supplemental Table 9- *Age Controlled Group Differences in Measures of Spindle and SWA*

| **Variable** | **INS (n= 119)** | **HS (n= 103)** | **Wald χ² (df= 1)** | **p** | **Coefficient** | **SE** | **p** | **95% Lower** | **Upper** | **Difference** |
| --- | --- | --- | --- | --- | --- | --- | --- | --- | --- | --- |
| **Fz** | | | | | | | | | | |
| **Event Detection** | | | | | | | | | | |
| Spindle Amplitude | 106.65 ± 2.30 | 113.52 ± 3.33 | 3.17 | .075 | -6.87 | 3.69 | .07 | -14.27 | 0.55 | - |
| SO Amplitude | 147.60 ± 3.58 | 166.07 ± 5.13 | 9.13 | .003 | **–18.47** | **6.15** | **.004** | **–31.06** | **–7.08** | **INS < HS** |
| **Power Spectral Analysis** | | | | | | | | | | |
| Relative SO Power | .63 ± .01 | .62 ± .01 | .024 | .877 | 0.002 | 0.012 | .892 | –0.022 | 0.026 | - |
| Absolute Sigma Power | 13.34 ± .65 | 12.56 ± .77 | 0.41 | .520 | 0.63 | 0.99 | .515 | -1.27 | 2.55 | - |
| Absolute SWA | 607.05 ± 30.95 | 733.72 ± 43.96 | 7.81 | .005 | **-139.57** | **51.78** | **.009** | **-243.51** | **-40.99** | **INS < HS** |
| Absolute SO Power | 494.66 ± 28.21 | 601.99 ± 40.85 | 6.15 | .013 | **–116.20** | **47.48** | **.015** | **–215.05** | **-25.60** | **INS < HS** |
| **Cz** | | | | | | | | | | |
| **Event Detection** | | | | | | | | | | |
| Spindle Amplitude | 96.97 ± 2.05 | 100.63 ± 1.90 | 1.60 | .206 | –3.66 | 2.91 | .202 | –9.59 | 2.13 | - |
| SO Amplitude | 131.68 ± 2.78 | 143.01 ± 3.14 | 6.72 | .010 | **-11.33** | **4.33** | **.007** | **-19.28** | **-2.91** | **INS < HS** |
| **Power Spectral Analysis** | | | | | | | | | | |
| Relative SO Power | .57 ± .01 | .58 ± .01 | 0.22 | .638 | -0.007 | 0.014 | .523 | –0.038 | 0.020 | - |
| Absolute Sigma Power | 16.67 ± .78 | 15.86 ± .85 | 0.29 | .591 | 0.60 | 1.11 | .582 | –1.57 | 2.73 | - |
| Absolute SWA | 468.41 ± 23.18 | 584.39 ± 32.50 | 11.68 | < .001 | **–125.89** | **38.12** | **.004** | **–195.50** | **–45.01** | **INS < HS** |
| Absolute SO Power | 371.79 ± 21.58 | 485.96 ± 38.61 | 9.03 | .003 | **-120.20** | **40.20** | **.007** | **-201.50** | **-41.25** | **INS < HS** |
| **Pz** | | | | | | | | | | |
| **Event Detection** | | | | | | | | | | |
| Spindle Density | 2.51 ± .07 | 3.50 ± .11 | 57.14 | <.001 | **–0.99** | **0.127** | **<.001** | **–1.23** | **-0.72** | **INS < HS** |
| Spindle Amplitude | 80.25 ± 1.79 | 89.93 ± 3.23 | 9.28 | .002 | **-9.68** | **3.25** | **.006** | **-16.04** | **-3.57** | **INS < HS** |
| SO Density | 4.76 ± .13 | 7.22 ± .35 | 45.59 | <.001 | **-2.46** | **0.382** | **<.001** | **-3.22** | **-1.74** | **INS < HS** |
| SO Amplitude | 112.54 ± 2.66 | 127.18 ± 4.20 | 10.00 | .002 | **-14.64** | **4.72** | **.002** | **-24.00** | **-5.83** | **INS < HS** |
| **Power Spectral Analysis** | | | | | | | | | | |
| Relative Sigma Power | .04 ± .002 | .04 ± .001 | 2.02 | .155 | -0.03 | 0.002 | .148 | –0.007 | 0.001 | - |
| Relative SWA | .73 ± .01 | .73 ± .01 | 0.01 | .920 | –0.001 | 0.009 | .926 | –0.019 | 0.017 | - |
| Relative SO Power | .61 ± .01 | .62 ± .01 | 0.23 | .632 | -0.007 | 0.014 | .634 | -0.033 | 0.021 | - |
| Absolute Sigma Power | 14.63 ± .78 | 13.86 ± .79 | 0.31 | .580 | 0.60 | 1.09 | .586 | –1.61 | 2.64 | - |
| Absolute SWA | 353.14 ± 18.71 | 424.83 ± 26.84 | 7.23 | .007 | **–79.99** | **29.84** | **.007** | **–138.21** | **–18.24** | **INS < HS** |
| Absolute SO Power | 311.64 ± 19.63 | 389.94 ± 32.51 | 6.04 | .014 | **-84.14** | **35.69** | **.020** | **-161.03** | **-18.82** | **INS < HS** |

Note. Values are reported as estimated marginals means (age controlled) ± standard error with bias-corrected confidence intervals. *p* = adjusted p; Significant results are bolded. Sigma power (11.25-16Hz), SWA (0.25-4Hz), SO power (0.25-1.25 Hz). INS = insomnia group; HS = healthy sleeper group. NREM2 + NREM3

Supplemental Table 10- *Age Controlled Sex Differences in Measures of Spindle and SWA*

| **Variable** | **Male (n= 74)** | **Female (n= 148)** | **Wald χ² (df= 1)** | **p** | **Coefficient** | **SE** | ***p*** | **95% Lower** | **Upper** | **Difference** |
| --- | --- | --- | --- | --- | --- | --- | --- | --- | --- | --- |
| **Fz** | | | | | | | | | | |
| **Event Detection** | | | | | | | | | | |
| Spindle Amplitude | 101.91 ± 3.35 | 118.25 ± 2.73 | 13.74 | <.001 | **-16.34** | **4.62** | **<.001** | **-25.34** | **-7.25** | **M < F** |
| SO Amplitude | 141.70 ± 4.94 | 171/97 ± 4.32 | 20.28 | <.001 | **–30.27** | **6.63** | **<.001** | **–42.40** | **-16.65** | **M < F** |
| **Power Spectral Analysis** | | | | | | | | | | |
| Relative SO power | .62 ± .01 | .63 ± .01 | 0.44 | .508 | -0.008 | .012 | .519 | -0.031 | 0.016 | - |
| Absolute Sigma power | 10.91 ± .70 | 14.01 ± .69 | 8.71 | .003 | **-3.07** | **1.04** | **.004** | **-5.24** | **-0.98** | **M < F** |
| Absolute SWA | 493.51 ± 33.21 | 747.38 ± 36.62 | 25.69 | <.001 | **–260.99** | **52.16** | **<.001** | **–359.53** | **-160.73** | **M < F** |
| Absolute SO power | 426.37 ± 40.23 | 599.90 ± 30.57 | 12.58 | < .001 | **–179.46** | **50.80** | **.002** | **–268.24** | **-84.09** | **M < F** |
| **Cz** | | | | | | | | | | |
| **Event Detection** | | | | | | | | | | |
| Spindle Amplitude | 88.39 ± 1.90 | 109.21 ± 1.98 | 55.33 | <.001 | **–20.82** | **2.87** | **<.001** | **–26.56** | **–15.08** | **M < F** |
| SO Amplitude | 122.39 ± 2.91 | 152.31 ± 2.98 | 48.27 | <.001 | **–29.92** | **4.34** | **<.001** | **–38.51** | **–21.28** | **M < F** |
| **Power Spectral Analysis** | | | | | | | | | | |
| Relative SO power | .57 ± .01 | .58 ± .01 | 0.44 | .507 | -0.009 | .014 | .523 | –0.038 | 0.020 | - |
| Absolute Sigma power | 13.44 ± .86 | 17.71 ± .75 | 12.85 | <.001 | **–4.24** | **1.23** | **.002** | **–6.70** | **–1.89** | **M < F** |
| Absolute SWA | 390.25 ± 27.73 | 584.26 ± 26.20 | 27.02 | <.001 | **–200.44** | **39.14** | **<.001** | **–277.40** | **-125.47** | **M < F** |
| Absolute SO power | 345.31 ± 46.46 | 461.13 ± 23.23 | 5.99 | .014 | **-121.95** | **50.67** | **.039** | **-216.14** | **-14.27** | **M < F** |
| **Pz** | | | | | | | | | | |
| **Event Detection** | | | | | | | | | | |
| Spindle Density | 2.97 ± .10 | 3.03 ± .08 | 0.28 | .598 | -0.06 | .128 | .610 | –0.33 | 0.16 | - |
| Spindle Amplitude | 77.46 ± 3.47 | 92.72 ± 2.20 | 14.07 | <.001 | **-15.26** | **4.15** | **.002** | **-22.81** | **-6.00** | **M < F** |
| SO Density | 5.89 ± .32 | 6.09 ± .22 | 0.25 | .617 | –0.197 | .406 | .634 | –0.985 | 0.593 | - |
| SO Amplitude | 106.02 ± 4.20 | 133.71 ± 3.27 | 26.62 | <.001 | **-27.70** | **5.43** | **<.001** | **-38.27** | **-17.28** | **M < F** |
| **Power Spectral Analysis** | | | | | | | | | | |
| Relative Sigma power | .04 ± .002 | .04 ± .001 | 7.79 | .005 | **0.006** | **.002** | **.008** | **0.002** | **0.011** | **M > F** |
| Relative SWA | .72 ± .01 | .74 ± .01 | 5.98 | .014 | **–0.024** | **.010** | **.016** | **–0.044** | **–0.006** | **M < F** |
| Relative SO power | .61 ± .01 | .62 ± .01 | 0.62 | .430 | -0.011 | .014 | .451 | -0.040 | 0.017 | - |
| Absolute Sigma power | 11.84 ± .80 | 15.48 ± .72 | 11.24 | <.001 | **-3.61** | **1.11** | **.002** | **-5.90** | **-1.32** | **M < F** |
| Absolute SWA | 275.29 ± 20.76 | 439.22 ± 22.05 | 29.16 | <.001 | **-168.01** | **31.25** | **<.001** | **–230.61** | **-104.35** | **M < F** |
| Absolute SO power | 270.53 ± 37.07 | 384.15 ± 21.29 | 8.15 | .004 | **-117.91** | **42.64** | **.018** | **-191.95** | **-33.29** | **M < F** |

Note. Values are reported as estimated marginals means (age controlled) ± standard error with bias-corrected confidence intervals. *p* = adjusted p; Significant results are bolded. Sigma power (11.25-16Hz), SWA (0.25-4Hz), SO power (0.25-1.25 Hz). M = male; F = female. NREM2 + NREM3

Supplemental Table 11- *Age Controlled Group by Sex Differences in Spindle and SWA Measures (Fz)*

| **(Fz)** | **Male INS**  **(n= 34)** | **Female INS**  **(n= 85)** | **Male HS**  **(n= 40)** | **Female HS**  **(n= 63)** | **Wald χ² (df= 3)** | **p** | **Coefficient** | **SE** | ***p*** | **95% Lower** | **Upper** | **Difference** |
| --- | --- | --- | --- | --- | --- | --- | --- | --- | --- | --- | --- | --- |
| **Event Detection** | | | | | | | | | | | | |
| Spindle | 98.68 ± 3.30 | 114.73 ± 2.90 | 105.18 ± 5.55 | 121.80 ± 4.51 | 20.94 | <.001 | -23.13 | 5.72 | <.001 | -33.69 | -11.43 | MINS < FHS |
| Amplitude |  |  |  |  |  |  | -16.06 | 4.49 | <.001 | -24.61 | -7.29 | MINS < FINS |
|  |  |  |  |  |  |  | 16.62 | 7.49 | .033 | 2.39 | 30.64 | MHS < FHS |
| SO | 132.31 ± 4.77 | 162.80 ± 4.70 | 151.06 ± 8.25 | 181.12 ± 7.13 | 37.45 | <.001 | 48.80 | 8.73 | <.001 | 32.40 | 67.06 | MINS < FHS |
| Amplitude |  |  |  |  |  |  | -18.32 | 8.41 | .021 | –36.04 | –2.99 | FINS < FHS |
|  |  |  |  |  |  |  | 30.49 | 6.89 | <.001 | 17.62 | 44.29 | MINS < FINS |
|  |  |  |  |  |  |  | -30.06 | 11.28 | .014 | -52.41 | -7.47 | MHS < FHS |
|  |  |  |  |  |  |  | **18.74** | **9.63** | **.054** | **0.53** | **38.50** | **MINS < MHS** |
| **Power Spectral Analysis** | | | | | | | | | | | | |
| Relative SO | .61 ± .02 | .64 ± .01 | .63 ± .01 | .62 ± .012 | 3.01 | .390 | - | - | - | - | - | - |
| Power |  |  |  |  |  |  |  |  |  |  |  |  |
| Absolute Sigma | 12.32 ± 1.08 | 13.80 ± .78 | 9.60 ± .90 | 14.30 ± 1.12 | 13.87 | .003 | -4.71 | 1.53 | .004 | -8.09 | -1.51 | MHS < FHS |
| Power |  |  |  |  |  |  | **2.73** | **1.41** | **.053** | **0.19** | **5.48** | **MINS > MHS** |
|  |  |  |  |  |  |  | 4.21 | 1.26 | <.001 | 2.01 | 7.30 | MHS < FINS |
| Absolute | 429.86 ± 32.65 | 685.41 ± 39.27 | 562.26 ± 53.92 | 828.85 ± 62.32 | 41.70 | <.001 | -399.00 | 74.29 | <.001 | -573.47 | -260.92 | MINS < FHS |
| SWA |  |  |  |  |  |  | -255.55 | 55.49 | <.001 | –374.94 | –137.67 | MINS < FINS |
| Power |  |  |  |  |  |  | 266.59 | 88.04 | <.001 | 108.08 | 432.41 | MHS < FHS |
|  |  |  |  |  |  |  | **143.45** | **74.28** | **.053** | **15.98** | **287.34** | **FINS < FHS** |
|  |  |  |  |  |  |  | -132.41 | 63.23 | .049 | -276.80 | -14.46 | MINS < MHS |
| Absolute | 345.29 ± 32.48 | 560.38 ± 36.07 | 508.48 ± 69.55 | 651.20 ± 51.91 | 32.78 | <.001 | 305.91 | 60.30 | <.001 | 201.20 | 432.44 | MINS < FHS |
| SO Power |  |  |  |  |  |  | 215.09 | 51.86 | <.001 | 115.36 | 329.78 | MINS < FINS |
|  |  |  |  |  |  |  | **163.19** | **75.94** | **.073** | **30.35** | **305.60** | **MINS < MHS** |

Note. Values are reported as estimated marginals means (age controlled) ± standard error with bias-corrected confidence intervals. Only significant post-hoc analyses are presented. *p* = adjusted p; Results in bold are FDR-corrected comparisons p > .05 but became significant after bias-corrected confidence intervals. Sigma power (11.25-16Hz), SWA (0.25-4Hz), SO power (0.25-1.25Hz). NREM2 + NREM3

Supplemental Table 12- *Age Controlled Group by Sex Differences in Spindle and SWA Measures (Cz)*

| **Cz** | **Male INS**  **(n= 34)** | **Female INS**  **(n= 85)** | **Male HS**  **(n= 40)** | **Female HS**  **(n= 63)** | ***Wald χ²* (df= 3)** | **p** | **Coefficient** | **SE** | ***p*** | **95% Lower** | **Upper** | **Difference** |
| --- | --- | --- | --- | --- | --- | --- | --- | --- | --- | --- | --- | --- |
| **Event Detection** | | | | | | | | | | | | |
| Spindle | 88.19 ± 2.55 | 106.67 ± 2.89 | 88.88 ± 2.77 | 111.96 ± 2.70 | 59.13 | <.001 | 23.76 | 3.81 | <.001 | 16.15 | 31.28 | MINS < FHS |
| Amplitude |  |  |  |  |  |  | –23.08 | 3.98 | <.001 | –31.26 | -15.47 | MHS < FHS |
|  |  |  |  |  |  |  | 18.48 | 3.88 | <.001 | 11.02 | 25.95 | MINS < FINS |
|  |  |  |  |  |  |  | 17.80 | 4.28 | <.001 | 9.37 | 25.95 | FINS > MHS |
| SO | 118.62 ± 3.66 | 145.83 ± 3.81 | 126.50 ± 4.43 | 159.04 ± 4.59 | 56.91 | <.001 | –40.42 | 5.95 | <.001 | 29.64 | 53.15 | MINS < FHS |
| Amplitude |  |  |  |  |  |  | –32.53 | 6.74 | <.001 | –45.78 | –19.01 | MHS < FHS |
|  |  |  |  |  |  |  | 27.21 | 5.22 | <.001 | 17.62 | 38.29 | MINS < FINS |
|  |  |  |  |  |  |  | 19.33 | 6.20 | .002 | 6.92 | 31.50 | FINS > MHS |
|  |  |  |  |  |  |  | -13.21 | 5.96 | .031 | -24.56 | -1.71 | FINS < FHS |
| **Power Spectral Analysis** | | | | | | | | | | | | |
| Relative SO Power | .55 ± .02 | .59 ± .01 | .59 ± .02 | .57 ± .01 | 3.82 | .281 | - | - | - | - | - | - |
| Absolute Sigma | 14.80 ± 1.29 | 17.50 ± .94 | 12.18 ± 1.14 | 18.00 ± 1.15 | 16.25 | .001 | 5.81 | 1.64 | <.001 | 2.88 | 8.92 | MHS < FHS |
| Power |  |  |  |  |  |  | -5.32 | 1.51 | .002 | -8.13 | -2.50 | MHS < FINS |
| Absolute | 331.07 ± 23.70 | 529.13 ± 29.46 | 453.83 ± 46.54 | 656.71 ± 43.40 | 53.01 | <.001 | 325.64 | 48.33 | <.001 | 232.59 | 433.77 | MINS < FHS |
| SWA |  |  |  |  |  |  | –127.58 | 52.15 | .016 | –224.17 | –30.01 | FINS < FHS |
| Power |  |  |  |  |  |  | 198.06 | 38.96 | <.001 | 126.56 | 280.86 | MINS < FINS |
|  |  |  |  |  |  |  | 122.76 | 52.28 | .036 | 28.66 | 224.89 | MINS < MHS |
|  |  |  |  |  |  |  | -202.88 | 67.45 | .005 | -322.39 | -80.38 | MHS < FHS |
| Absolute | 252.38 ± 23.23 | 424.14 ± 27.75 | 438.27 ± 85.57 | 508.86 ± 39.33 | 41.69 | <.001 | –256.48 | 45.63 | <.001 | –354.17 | -167.74 | MINS < FHS |
| SO Power |  |  |  |  |  |  | -171.78 | 38.83 | <.001 | -245.92 | -105.62 | MINS < FINS |
|  |  |  |  |  |  |  | **-185.88** | **89.00** | **.114** | **-399.30** | **-18.72** | **MINS < MHS** |

Note. Values are reported as estimated marginals means (age controlled) ± standard error with bias-corrected confidence intervals. Only significant post-hoc analyses are presented. *p* = adjusted p; Results in bold are FDR-corrected comparisons p > .05 but became significant after bias-corrected confidence intervals. Sigma power (11.25-16Hz), SWA (0.25-4Hz), SO power (0.25-1.25Hz). NREM2 + NREM3

Supplemental Table 13- *Age Controlled Group by Sex Differences in Spindle and SWA Measures (Pz)*

| **(Pz)** | **Male INS**  **(n= 34)** | **Female INS**  **(n= 85)** | **Male HS**  **(n= 40)** | **Female HS**  **(n= 63)** | ***Wald χ²* (df= 3)** | **p** | **Coefficient** | **SE** | ***p*** | **95% Lower** | **Upper** | **Difference** |
| --- | --- | --- | --- | --- | --- | --- | --- | --- | --- | --- | --- | --- |
| **Event Detection** | | | | | | | | | | | | |
| Spindle  Density | 2.59 ± .10 | 2.49 ± .08 | 3.37 ± .16 | 3.60 ± .15 | 58.99 | <.001 | 0.77 | 0.19 | <.001 | 0.384 | 1.151 | MINS < MHS |
|  |  |  |  |  |  |  | 1.00 | 0.17 | <.001 | 0.697 | 1.359 | MINS < FHS |
|  |  |  |  |  |  |  | -0.88 | 0.18 | <.001 | -1.228 | -0.526 | FINS < MHS |
|  |  |  |  |  |  |  | -1.11 | 0.17 | <.001 | -1.45 | -0.792 | FINS < FHS |
| Spindle | 72.04 ± 1.84 | 88.13 ± 2.34 | 82.77 ± 6.28 | 97.23 ± 3.54 | 50.71 | <.001 | 25.20 | 4.04 | <.001 | 17.85 | 33.71 | MINS < FHS |
| Amplitude |  |  |  |  |  |  | -9.11 | 4.00 | .028 | -17.17 | -1.34 | FINS < FHS |
|  |  |  |  |  |  |  | 16.09 | 3.06 | <.001 | 10.30 | 22.27 | MINS < FINS |
| SO Density | 4.58 ± .19 | 4.89 ± .14 | 7.19 ± .58 | 7.28 ± .44 | 46.97 | <.001 | 2.39 | .45 | <.001 | 1.458 | 3.255 | FINS < FHS |
|  |  |  |  |  |  |  | -2.60 | .61 | .002 | -3.89 | -1.48 | MINS < MHS |
|  |  |  |  |  |  |  | -2.70 | .50 | <.001 | -3.72 | -1.72 | MINS < FHS |
|  |  |  |  |  |  |  | 2.30 | .62 | .004 | 1.076 | 3.538 | FINS < MHS |
| SO | 100.06 ± 3.09 | 125.80 ± 3.55 | 112.22 ± 7.37 | 141.80 ± 5.35 | 55.14 |  | –41.74 | 6.23 | <.001 | –55.33 | –30.97 | MINS < FHS |
| Amplitude |  |  |  |  |  |  | 16.00 | 6.23 | .012 | 3.82 | 28.85 | FINS < FHS |
|  |  |  |  |  |  |  | 29.58 | 9.64 | .005 | 9.92 | 47.84 | MHS < FHS |
|  |  |  |  |  |  |  | -25.74 | 4.84 | <.001 | -35.74 | -16.43 | MINS < FINS |
| **Power Spectral Analysis** | | | | | | | | | | | | |
| Relative Sigma  Power | .04 ± .003 | .03 ± .002 | .04 ± .002 | .04 ± .002 | 12.47 | .006 | 0.006 | 0.00 | .025 | 0.001 | 0.011 | MHS > FHS |
|  |  |  |  |  |  |  | -0.009 | 0.00 | .003 | -0.015 | -0.003 | FINS < MHS |
| Relative SWA | .72 ± .01 | .74 ± .01 | .71 ± .013 | .74 ± .01 | 6.17 | .104 | - | - | - | - | - | - |
| Relative SO Power | .59 ± .02 | .63 ± .01 | .63 ± .02 | .62 ± .01 | 3.82 | .282 | - | - | - | - | - | - |
| Absolute | 12.58 ± 1.04 | 15.54 ± .99 | 11.13 ± 1.22 | 15.41 ± 1.02 | 11.60 | .009 | -4.27 | 1.67 | .011 | -7.57 | -1.02 | MHS < FHS |
| Sigma |  |  |  |  |  |  | 2.96 | 1.39 | .040 | 0.41 | 5.57 | MINS < FINS |
| Power |  |  |  |  |  |  | 4.41 | 1.60 | .014 | 1.67 | 7.57 | MHS < FINS |
| Absolute | 240.20 ± 15.57 | 403.10 ± 24.31 | 313.46 ± 36.26 | 486.73 ± 36.72 | 54.12 | <.001 | –246.53 | 41.23 | <.001 | -330.71 | -170.69 | MINS < FHS |
| SWA |  |  |  |  |  |  | **83.63** | **43.14** | **.061** | **2.52** | **161.16** | **FINS < FHS** |
| Power |  |  |  |  |  |  | -162.90 | 30.05 | <.001 | -226.65 | -110.70 | MINS < FINS |
|  |  |  |  |  |  |  | -89.65 | 42.00 | .041 | -166.72 | -14.02 | MHS < FINS |
|  |  |  |  |  |  |  | 173.27 | 54.03 | .004 | 60.47 | 293.68 | MHS < FHS |
| Absolute | 207.14 ± 18.73 | 357.55 ± 25.74 | 334.13 ± 68.68 | 418.54 ± 35.03 | 39.42 | <.001 | 211.40 | 40.80 | <.001 | 134.10 | 293.78 | MINS < FHS |
| SO Power |  |  |  |  |  |  | 150.41 | 33.46 | <.001 | 80.40 | 216.07 | MINS < FINS |
|  |  |  |  |  |  |  | 126.99 | 72.35 | .156 | 8.47 | 271.17 | MINS < MHS |

Note. Values are reported as estimated marginals means (age controlled) ± standard error with bias-corrected confidence intervals. Only significant post-hoc analyses are presented. *p* = adjusted p; Results in bold are FDR-corrected comparisons p > .05 but became significant after bias-corrected confidence intervals. Sigma power (11.25-16Hz), SWA (0.25-4Hz), SO power (0.25-1.25Hz). NREM2 + NREM3
